# Lithotomy

**Published:** 1829-03

**Authors:** 


### LITHOTOMY.

*Extraordinary Case of a large oblong Calculus impacted in the Neck of the Bladder and Prostatic Portion of the Urethra, for the Extraction of which the Patient underwent two different Operations the same day.* By F. Blandin, second Surgeon of the Hôpital Beaujon.

A boy, fifteen years old, was admitted into the Hôpital Beaujon, on the 1st December, 1828, who stated to M. Blandin that during the last five years he had habitually suffered considerable pain at the bottom of the abdomen above the bladder; and that its intensity was always increased by the exertions of walking, by the evacuation of the feces, and by micturition. The pain extended along the whole course of the urethra to the glans, in which part, indeed, it was most acute. The day of admission, the penis was in a constant state of semierection, and the patient was frequently prompted, as it were instinctively, to grasp the part forcibly with his hand, in order to diminish the sensation of pain, from which he seemed to be scarcely ever free. His urine passed from him involuntarily and guttatim; the anus was encircled by a prominent hemorrhoidal band. He was frequently affected with diarrhœa, extremely emaciated, and his countenance indicated great prostration and suffering.

When Mr. B. introduced the catheter into the urethra, it occasioned such excessive pain that the bare idea of this operation threw the patient into a condition resembling the incipient stage of an intermittent fever. It was stopped, as soon as it arrived at the neck of the bladder, by a solid mass which appeared to be quite immoveably fixed in that part, and returned a distinct sound when struck. From these circumstances M. Blandin was of opinion that a very large calculus was situated in the bladder, in such a manner as to preclude the entrance to this viscus, by pressing its neck towards the perineum. This opinion was immediately afterwards strengthened, when, on introducing his finger into the rectum, he could distinctly feel the calculus through the parts interposing between the finger and the bottom of the bladder, which it had considerably depressed.

Accordingly, M. Blandin forthwith announced that it was his intention to adopt in this case the *high operation,* since he conceived the stone to be too large to allow of extraction by the lateral mode.

On the morrow (2d December,) Professor Marjolin, who examined the patient, coincided with M. B. in his general views of the case ; fully admitted the necessity, likewise, of operating above the pubes; and pronounced that the calculus was situated transversely, and in contact with both the tuberosities of the ischia.

The patient was ordered to bathe the whole body; to have an emollient clyster, a cataplasm to his abdomen; and to drink abundantly of almond emulsion.

This treatment was continued from the 1st to the 7th December, which was the day chosen for the operation.

After again verifying the existence of a stone in the bladder, by the introduction of the sound, &c. M. Blandin, in the presence of M. Marjolin, several other practitioners of eminence, and many students, proceeded to operate in the following manner:

He commenced by injecting some decoction of mallows through the catheter into the bladder, which admitted but a small quantity of the injection, owing to its contracted state. The catheter was now withdrawn, and an assistant was employed to press the sides of the urethra together, in order to prevent the fluid contents of the bladder from escaping through that canal. M. B. then, with a convex bistoury, divided the integuments of the hypogastrium in their centre, from above downwards, for the space of about four inches above the pubes. The linea alba was first divided near the pubes, whence the forefinger of the left hand being carried behind and upwards, the section was completed in that direction from within by the bistouri boutonné. Then the same finger being passed down through the wound to the anterior parietes of the bladder, was used to direct thither a straight pointed bistoury, with which this viscus was opened. But its muscular fibres were so much thickened by an almost constant state of contraction for some years, that several strokes of the bistoury were required to divide it sufficiently to allow the urine to escape freely through the wound. As soon as this was accomplished, a hook was inserted into its upper part, and a pair of small forceps carefully introduced by the guidance of a finger into the bladder, where they instantly reached and laid hold of the calculus, but could neither be made to retain their grasp, nor even so much as to move it in situ.

M. Blandin, therefore, withdrawing his instruments, examined the parts with his finger, and having discovered a middling-sized calculus which was lodged at the upper part of the bladder, he immediately extracted it with the forceps. He again endeavoured to remove the large calculus situated at the lowest part of the bladder, but to no purpose, though his efforts were seconded by those of an assitant, who attempted to elevate it by his finger in the rectum. The forceps, despite his utmost strength, could not retain their hold, and only brought away a few fragments of the stone; and, on again introducing his finger into the wound, M. B. found that the stone was quite as immoveable as at first, and also discovered that its small extremity alone projected into the cavity of the bladder, whilst the greater portion of it was contained in a sort of sac, in which it was closely confined.

After that M. Marjolin and the other surgeons present had verified the above circumstances by personal examination, M. Blandin successively introduced a finger into the rectum, and a sound into the urethra, and thus tried, but in vain, to propel the calculus upwards and backwards. Then, by introducing a blunt instrument into the bladder, and carefully insinuating its extremity between the calculus and the sac which contained it, he endeavoured to raise and disengage the stone by using the instrument as a lever of the first kind, of which his finger formed the fulcrum. But although aided in this attempt by an assistant, who, having passed his finger up the rectum, employed considerable force simultaneously to effect the same purpose, it was only a little loosened, and its extrication from the artificial cavity in which it was impacted seemed no more likely to be achieved now, than at the commencement of the operation.

At this period of his exertions, M. Blandin, after consulting anew with M. Marjolin and the other experienced surgeons present, concluded that the exhausted state of the patient was unfavorable for the continuance of these fatiguing and unsatisfactory manœuvres; and he therefore determined to cut at once through the centre of the perineum, and to divide the neck of the bladder, with the adjoining portion of the rectum, in the hope that he might be thus enabled to thrust back the calculus.

M. Blandin, in order to effect this, passed the left forefinger up the anus, until it came in contact with the indurated tumor which depressed the bladder. Guided by this finger, he carried up to its extremity, with his right hand, a straight pointed bistoury, the point of which being then simultaneously elevated and withdrawn, effected in a single time, in a central direction, and with the greatest facility, the division of the neck of the bladder, the prostate, the forepart of the anus, and adjoining portion of the perineum. In this manner the stone was laid completely bare, and examined by the finger; and M. B. succeeded, but not without difficulty, in passing a large pair of forceps between the calculus and the cul-de-sac in which it was lodged. He renewed his endeavours to loosen and thrust it up towards the first incision; but, finding still an obstacle in this direction, he changed his plan, and, by employing an assistant to pass his finger through the wound of the hypogastrium, and to press the calculus from above downwards, he at length succeeded in pulling out the stone with the forceps through the opening in the perineum. It was an oblong calculus, hard, and of a crystalline appearance; it was nearly two and a half inches in length; its smallest diameter measured about an inch and a half, and it weighed two ounces.

Though the operation was long and fatiguing, yet the patient lost very little blood, and, as soon as it was terminated, he was conveyed back to his bed.

Symptoms of peritoneal inflammation soon came on, which resisted the treatment employed, and the child died the third day from the operation.

From the appearances detected on dissection, it was very evident that the patient had died from infiltration of urine in the cellular tissue, and from inflammation of the peritoneum. M. Blandin suggests that this fact alone is important, as it militates against the opinion of those who declare "that peritonitis never occurs from causes acting upon its external surface." He candidly expresses his apprehension that the closure of the wound in the hypogastrium by adhesive bandages had, to a certain extent, favored the urinous infiltration. The efforts which were made by nature, after the operation, to discharge the urine by the wound, confirm this opinion. This disadvantage was quickly detected, and the bandages were removed, and a seton was passed between the wounds in the hypogastrium and the perineum, to facilitate the exit of the urine. The incision in the perineum had left untouched the neck of the bladder, and hence the difficulty of an escape of urine from this part.

